# Supplementary material for: Segmentation-Less, Automated, Vascular Vectorization
Source: PLoS Comput Biol. 2021 Oct 8;17(10):e1009451. doi: 10.1371/journal.pcbi.1009451 (PMC8528315; doi:10.1371/journal.pcbi.1009451)
Supplement: S1 Fig — (PDF) [file pcbi.1009451.s001.pdf]

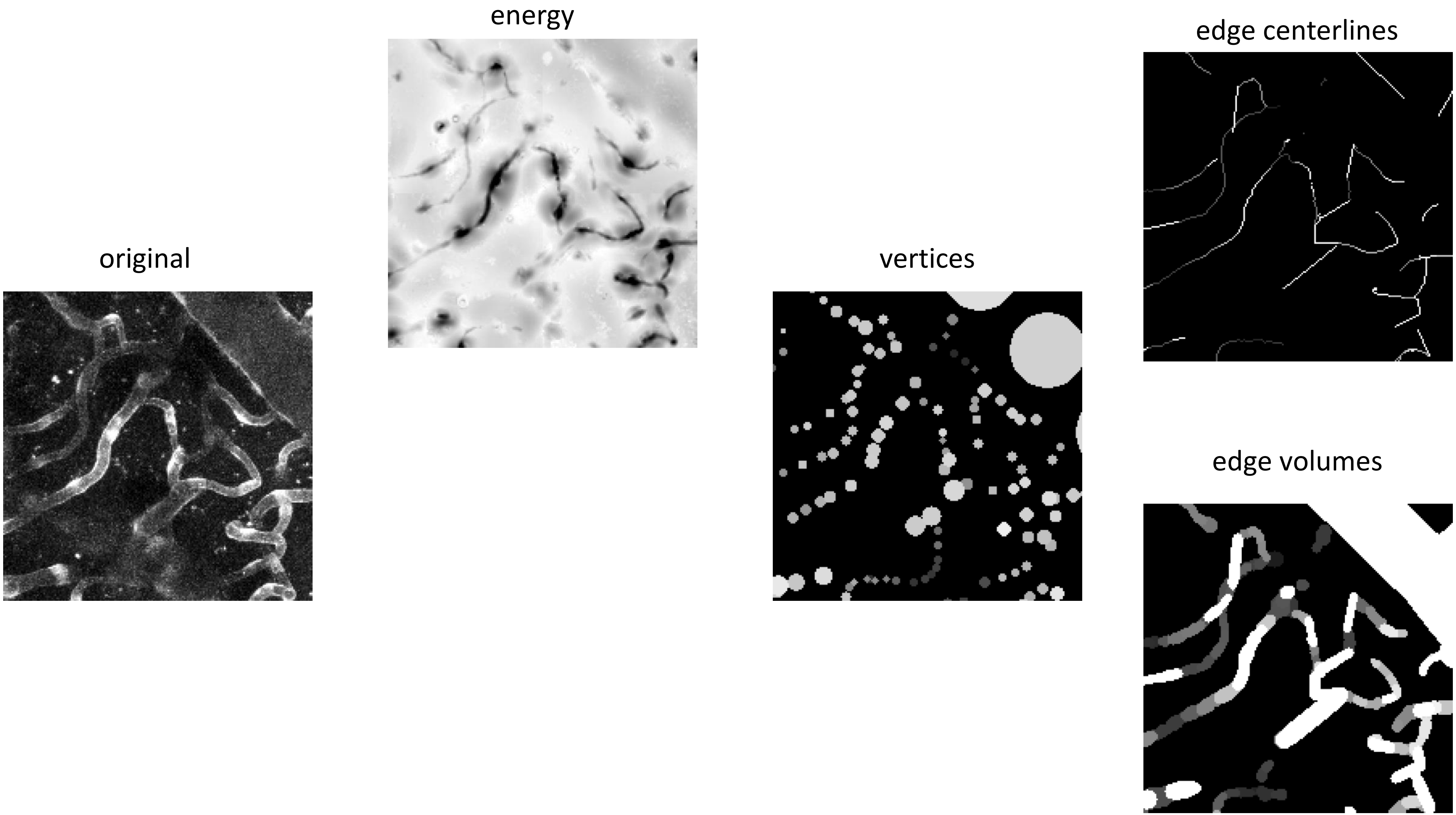

S1_Fig: Example projections of original two-photon image, intermediate outputs, and vector renderings of manually assisted SLAVV applied to Image 2. The vectors extracted from endothelial label are similar to those from lumen label (see Fig 2A).
